# Evaluation of Sample Pooling for SARS-CoV-2 Detection in Nasopharyngeal Swab and Saliva Samples with the Idylla SARS-CoV-2 Test

**DOI:** 10.1128/Spectrum.00996-21

**Published:** 2021-11-10

**Authors:** Paul Hofman, Maryline Allegra, Myriam Salah, Jonathan Benzaquen, Virginie Tanga, Olivier Bordone, Julien Fayada, Elodie Long-Mira, Sandra Lassalle, Elisabeth Lanteri, Virginie Lespinet-Fabre, Patrick Brest, Baharia Mograbi, Charlotte Maniel, Jacques Boutros, Sylvie Leroy, Simon Heeke, Véronique Hofman, Charles-Hugo Marquette, Marius Ilié

**Affiliations:** a Laboratory of Clinical and Experimental Pathology, Centre Hospitalier Universitaire de Nice, FHU OncoAge, Université Côte d'Azur, Nice, France; b Hospital-Related Biobank (BB-0033-00025), Centre Hospitalier Universitaire de Nice, FHU OncoAge, Université Côte d'Azur, Nice, France; c Team 4, Institute of Research on Cancer and Aging, CNRS INSERM, Centre Antoine-Lacassagne, Université Côte d'Azur, Nice, France; d Department of Pulmonary Medicine and Oncology, Centre Hospitalier Universitaire de Nice, FHU OncoAge, Université Côte d'Azur, Nice, France; e Department of Thoracic/Head and Neck Medical Oncology, The University of Texas MD Anderson Cancer Center, Houston, Texas, USA; Quest Diagnostics Nichols Institute

**Keywords:** COVID-19, pooling, SARS-CoV-2, Idylla test

## Abstract

Due to increased demand for testing, as well as restricted supply chain resources, testing for severe acute respiratory syndrome coronavirus 2 (SARS-CoV-2) infection continues to face many hurdles. Pooling several samples has been proposed as an alternative approach to address these issues. We investigated the feasibility of pooling nasopharyngeal swab (NPS) or saliva samples for SARS-CoV-2 testing with a commercial assay (Idylla SARS-CoV-2 test; Biocartis). We evaluated the 10-pool and 20-pool approaches for 149 subjects, with 30 positive samples and 119 negative samples. The 10-pool approach had sensitivity of 78.95% (95% confidence interval [CI], 54.43% to 93.95%) and specificity of 100% (95% CI, 71.51% to 100%), whereas the 20-pool approach had sensitivity of 55.56% (95% CI, 21.20% to 86.30%) and specificity of 100% (95% CI, 25% to 100%). No significant difference was observed between the results obtained with pooled NPS and saliva samples. Given the rapidity, full automation, and practical advantages of the Idylla SARS-CoV-2 assay, pooling of 10 samples has the potential to significantly increase testing capacity for both NPS and saliva samples, with good sensitivity.

**IMPORTANCE** To control outbreaks of coronavirus disease 2019 (COVID-19) and to avoid reagent shortages, testing strategies must be adapted and maintained for the foreseeable future. We analyzed the feasibility of pooling NPS and saliva samples for SARS-CoV-2 testing with the Idylla SARS-CoV-2 test, and we found that sensitivity was dependent on the pool size. The SARS-CoV-2 testing capacity with both NPS and saliva samples could be significantly expanded by pooling 10 samples; however, pooling 20 samples resulted in lower sensitivity.

## INTRODUCTION

The control of the coronavirus disease 2019 (COVID-19) pandemic requires huge efforts to allow widespread screening for severe acute respiratory syndrome coronavirus 2 (SARS-CoV-2) in the general population and to enable testing and contact tracing. In addition, these measures must include the ability to detect known virus variants of SARS-CoV-2 promptly and in large numbers, as well as immediately identifying the emergence of novel variants that emerge throughout the pandemic (1).

Several challenges continue to hamper the testing for SARS-CoV-2 infection promptly, accurately, and in large numbers, due in part to the rapid increase in cases and the complexity of the testing procedure but also to the limited resources in the supply chain, from collection devices to testing reagents (2). Therefore, limited laboratory capacity worldwide, particularly in Africa, South America, and some parts of Asia (3, 4), has hindered access to testing for SARS-CoV-2 and has delayed results. In order to overcome these issues and to increase testing throughput, pooling of multiple samples has been proposed as a strategy (5). The pooling strategy is considered a practical and effective method to analyze large quantities of samples without significantly loss of performance, especially when it comes to centralized testing models with automated systems. Several teams have recently reported successful pooling of SARS-CoV-2 samples (6–11). However, those reports were limited to geographic areas such as the United States or South Korea. In addition, in the United States an emergency use authorization was required by the Food and Drug Administration for testing pooled samples for asymptomatic screening, offering a regulatory basis for the selection of the testing methods and strategies that is lacking in other countries. Some of the methods used were laboratory-developed real-time (RT)-PCR tests, often requiring up to hundreds of samples to be grouped in batches to be tested in parallel. One of the consequences was a prolonged delay for the results to be obtained, i.e., 6 to 24 h after nasopharyngeal swab (NPS) specimen collection but possibly even longer, depending on the local conditions and the organization of sample workflows and technologies. We previously validated a fast and accurate ready-to-use RT-PCR assay using the Idylla platform (Biocartis, Mechelen, Belgium) (12), which had 100% positive and negative agreement with standard-of-care RT-PCR tests. However, data are still limited regarding the best strategy for detecting SARS-CoV-2 cases by pooling samples and assessing how they influence the analytical precision of the RT-PCR analysis (2, 8, 11, 13–20). Therefore, it is necessary to continue assessing the potential of pooling NPS and saliva samples to increase SARS-CoV-2 testing capacity in clinical laboratories, especially with commercially available assays. In this study, we aimed to investigate the feasibility of two strategies for pooling NPS and saliva samples for SARS-CoV-2 testing with the Idylla commercial assay.

## RESULTS

### Subjects.

NPS and saliva samples were collected from 149 subjects referred (i) by their attending physician because of recent (≤2 weeks) symptoms of COVID-19 or (ii) by the contact-tracing staff of the French public health insurance, since they were considered close contacts of a laboratory-confirmed COVID-19 case.

### Pools of 10 samples.

There was a contingency between the variables, with a significant correlation between the SARS-Cov-2 individual results and the results obtained with the pooled samples (*P < *0.0001) (Table 1). In our study, the analytical sensitivity and specificity for pools of 10 samples were equal to 78.95% (95% confidence interval [CI], 54.43% to 93.95%) and 100% (95% CI, 71.51% to 100%), respectively. The estimated accuracy was 98.11% (95% CI, 85.08% to 100%). The absolute percentage of agreement between the two groups was 86.66%. There was substantial agreement between the two groups (*k *= 0.733).

**TABLE 1:** Agreement contingency table used to analyze the association between the individual SARS-Cov-2 results and the results obtained with the pooled samples (1/10 samples)

Individual assay result	No. (%) with pooled assay result of:		
	Negative	Positive	Total
Negative	11 (36.7)	0 (0)	11 (36.7)
Positive	4 (13.3)	15 (50)	19 (63.3)
Total	15 (50)	15 (50)	30 (100)

**FIG 1:**
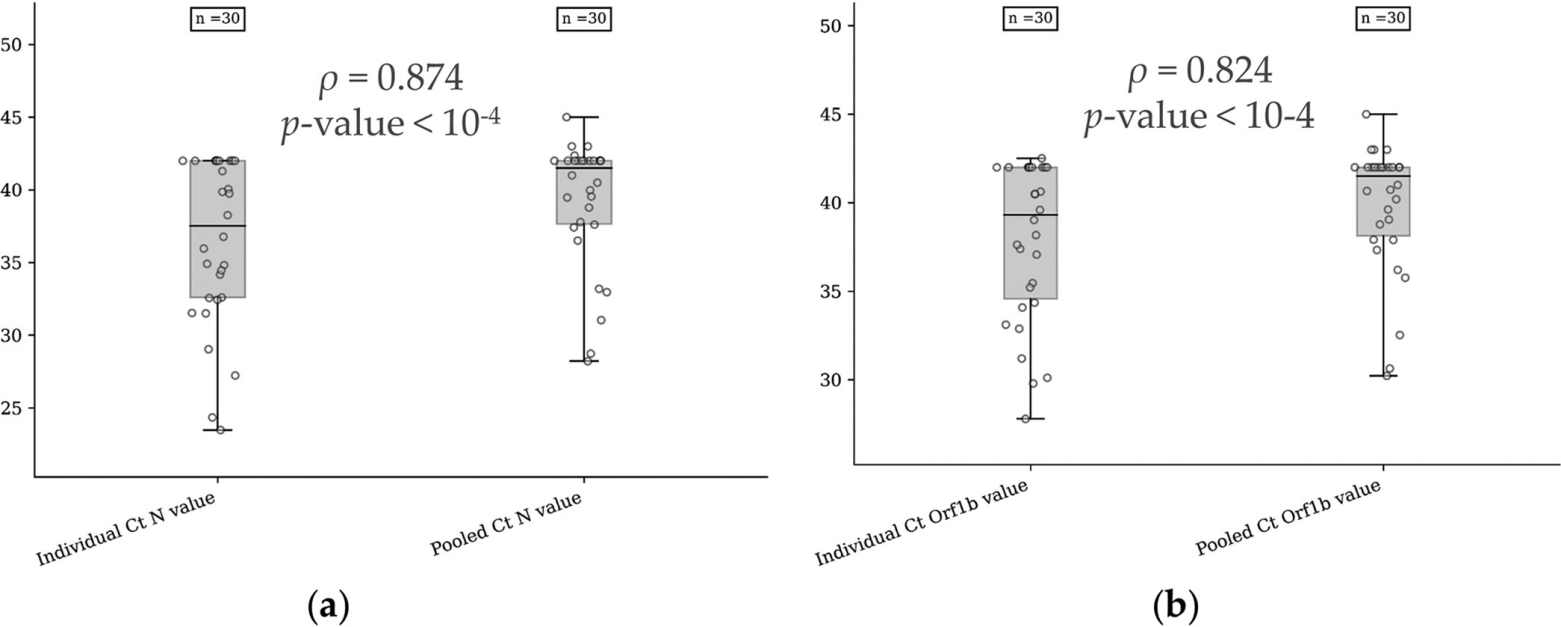
Distribution of the *C_T_* values for the N gene (a) and the Orf1b gene (b) measured with the SARS-CoV-2 Idylla test with individual samples or pooled samples (1/10 samples). The Pearson product-moment correlation coefficient was used to assess linear dependence between the *C_T_* values.

The median N cycle threshold (*C_T_*) value for the individual positive samples was 37.52 (range, 23.48 to 42.00; interquartile range [IQR], 9.41 [95% CI, 34.42 to 38.58]), whereas the median N *C_T_* value for the pooled samples was 41.50 (range, 28.22 to 45; IQR, 34 [95% CI, 37.67 to 40.87]). The median open reading frame 1b (Orf1b) *C_T_* value for the individual positive samples was 39.31 (range, 27.79 to 42.51; IQR, 7.43 [95% CI, 36.26 to 39.53]), while it was equal to 41.50 for the pooled samples (range, 30.22 to 45; IQR, 3.87 [95% CI, 38.47 to 41.15]). There was a strong positive linear correlation between the *C_T_* values for the N gene (ρ = 0.874, *P < *10^−4^) (Fig. 1a) or the Orf1b gene (ρ = 0.824, *P < *10^−4^) (Fig. 1b) for individual and pooled samples.

No significant difference was observed between the results obtained with pooled NPS or saliva samples (*P = *0.202). However, the comparison between the *C_T_* values (N or Orf1b) for the NPS and saliva samples showed significantly higher values in saliva samples than in NPS samples (median ± standard deviation [SD] N *C_T_* values: NPS, 35.33 ± 4.925; saliva, 40.94 ± 3.05 [*P = *0.0034]; median ± SD Orf1b *C_T_* values: NPS, 36.36 ± 4.23; saliva, 41.25 ± 2.35 [*P = *0.0021]).

### Pools of 20 samples.

The frequency distribution of the variables was independent, as there was no significant association between the individual SARS-Cov-2 results and the results obtained with the pooled samples (*P* = 1) (Table 2). In our study, the analytical sensitivity and specificity for pools of 20 samples were equal to 55.56% (95% CI, 21.20% to 86.30%) and 100% (95% CI, 25% to 100%), respectively. The estimated accuracy was 96% (95% CI, 63.24% to 100%). The absolute percentage of agreement between the two groups was 60%. There was a slight agreement between the two groups (*k *= 0.2).

**TABLE 2:** Agreement contingency table used to analyze the association between the individual SARS-Cov-2 results and the results obtained with the pooled samples (1/20 samples)

Individual assay result	No. (%) with pooled assay result of:		
	Negative	Positive	Total
Negative	1 (10)	0 (0)	1 (10)
Positive	4 (40)	5 (50)	9 (90)
Total	5 (50)	5 (50)	10 (100)

**FIG 2:**
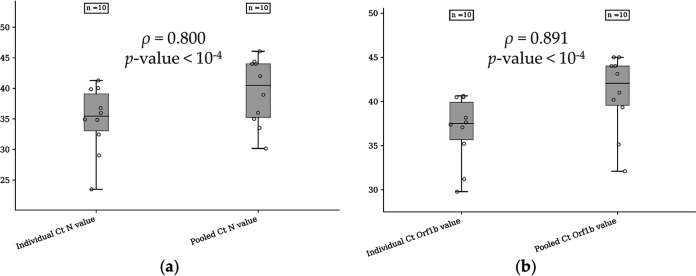
Distribution of the *C_T_* values for the N gene (a) and the Orf1b gene (b) measured with the SARS-CoV-2 Idylla test with individual or pooled samples (1/20 samples). The Pearson product-moment correlation coefficient was used to assess linear dependence between the *C_T_* values.

The median N *C_T_* value for the individual positive samples was 35.44 (range, 23.48 to 41.29; IQR, 6.04 [95% CI, 31.16 to 38.56]), whereas the median N *C_T_* value for the pooled samples was 40.48 (range, 30.16 to 46.05; IQR, 8.74 [95% CI, 35.69 to 43.11]). The median Orf1b *C_T_* value for the individual positive samples was 37.51 (range, 29.79 to 40.63; IQR, 4.22 [95% CI, 34.24 to 39.36]), whereas that for the pooled samples was equal to 42.06 (range, 32.10 to 45; IQR, 4.44 [95% CI, 37.92 to 43.85]). There was a strong positive linear correlation between the *C_T_* values for the N gene (ρ = 0.800, *P < *10^−4^) (Fig. 2a) or the Orf1b gene (ρ = 0.891, *P < *10^−4^) (Fig. 2b) for individual and pooled samples.

No significant difference was observed between the results obtained for pooled NPS or saliva samples (*P = *0.206). In addition, the comparison between the *C_T_* values (N or Orf1b) for the NPS and saliva samples showed higher values in saliva samples than in NPS samples (median ± SD N *C_T_* values: NPS, 36.13 ± 4.12; saliva, 42.68 ± 3.91 [*P = *0.296]; median ± SD Orf1b *C_T_* values: NPS, 38.14 ± 3.99; saliva, 43.64 ± 1.77 [*P = *0.210]).

## DISCUSSION

These preliminary results demonstrated that the 10-pool approach was sensitive and accurate for the detection of SARS-CoV-2 infection in both NPS and saliva pooled samples from symptomatic and asymptomatic individuals, while the pooling of 20 samples showed a drastic decrease in sensitivity. In this setting, the prevalence of the disease was low, ∼1%.

Since the beginning of the COVID-19 pandemic, various pooling strategies have been developed (6–11). The effect of pooling on sensitivity varied among previous studies and was dependent on the pool size, platform, and assay employed (6–11). Prior studies found that, as the pool size increased (range, 2 to 20 samples), sensitivity decreased and *C_T_* values for target genes increased (6–11).

In our study, the approach of pooling 20 samples (1 positive sample/20 samples) showed a loss of sensitivity within a 1% positive rate, while the approach of pooling 10 samples (1 positive sample/10 samples) showed sensitivity and specificity values similar to those in prior studies (2). Currently, there are limited data on assessment of the optimal pool size (21). The pool size should be selected according to the disease prevalence to save tests and thus to be cost- and time-effective. In addition, some studies did not include samples with *C_T_* values above 35, which could have had an impact on the reported overall excellent sensitivity (7). The strength of our study resides in the fact that we analyzed consecutive pools of samples, which simulated the real-life situation, including positive samples with low viral loads.

In each pooling approach in our study, false-negative results occurred, all in saliva samples with *C_T_* values of >35, suggesting a potential decrease in sensitivity for low viral loads, as reported previously (8, 9). False-negative results are of concern because infected individuals might not be isolated and could infect others (22). Although special precautions should be taken with RT-PCR false-negative results (22), it is also known that the majority of positive results obtained with just one targeted gene and with *C_T_* values of >35 correspond to nonviable/noninfectious particles that are nonetheless detected by RT-PCR (23). In addition, we did not observe false-positive results in our study, even for samples with *C_T_* values of >35.

We also evaluated pooling of saliva samples. There was no significant difference between the results obtained with pooled NSP samples and saliva samples. We and others previously showed that saliva samples were acceptable samples for SARS-CoV-2 detection, with the added advantages of allowing for self-collection and not requiring trained nursing staff members, sialagogic drugs, or particular constraints (such as fasting); their acceptability makes them ideal for institutionalized elderly people or for children (24–26). While studies on the subject are scarce, the approach of combining pooling with saliva collection could further expand the availability of testing (11, 19). However, variations in *C_T_* values between individual and pooled saliva samples appeared to be more evident than with NPS samples, as shown previously (6, 11). This could be attributed to possible inhibition in saliva samples with greater mucus contents (11). Further studies using a larger sample set will be needed to determine the effect of pooling on saliva virus loads.

The main limitations of our study were the retrospective design and the inclusion of a smaller sample size for cases. However, even with this design, our results proved that the approach of pooling 10 samples is sensitive and concordant with the individual RT-PCR results for both NPS and saliva samples. In conclusion, we demonstrated that the pooling of 10 samples has the potential to significantly increase SARS-CoV-2 testing capacity with both NPS and saliva samples with good sensitivity, whereas pooling of 20 samples shows significantly lower sensitivity.

## MATERIALS AND METHODS

### Patients and samples.

This retrospective study was performed with a cohort of 149 consecutive subjects who were enrolled in a large prospective study (ClinicalTrial.gov registration no. NCT04418206) conducted at the University Côte d’Azur COVID-19 Biobank (BB-0033-00025, Pasteur Hospital, Nice, France) (27). The subjects volunteered at the Nice-Côte d’Azur Metropolis community-based COVID-19 center (Nice, France), which was accessible to the general population for screening for a period of 22 weeks (21 September 2020 to 23 March 2021) (25, 26). The mean age ± SD was 43 ± 15 years; 83 subjects were female and 66 were male, as described previously (25, 26).

NPS or saliva samples were collected randomly from the subjects. The interval between symptom onset and testing was 3.6 ± 2.6 days, and most participants (107/149 subjects [72%]) were sampled in the early stage of the disease, i.e., within 4 days after symptom onset. Of those subjects, 30 (20%) had positive RT-PCR results with one of the sampling techniques and thus were diagnosed as having COVID-19. Informed consent was obtained from all subjects involved in the study.

For SARS-CoV-2 detection, the Idylla SARS-CoV-2 kit was used on the Idylla platform (Biocartis), as reported previously (12). A positive or negative result was determined according to the instructions for use of the Idylla SARS-CoV-2 test. Pool screening was performed using this assay and targeting the same target as for individual samples. All samples were stored at −80°C at the University Côte d’Azur COVID-19 Biobank (BB-0033-00025, Louis Pasteur Hospital, Nice, France) prior to analysis (27). The sponsor of the study was Nice University Hospital. The study was conducted according to the guidelines of the Declaration of Helsinki and was approved by the Institutional Review Board Sud Méditerranée V of Centre Hospitalier Universitaire de Nice (registration no. 20.04014.35208 [date of approval, 22 April 2020]), with SHAM liability insurance (no. 159087).

### Sample pooling.

**FIG 3:**
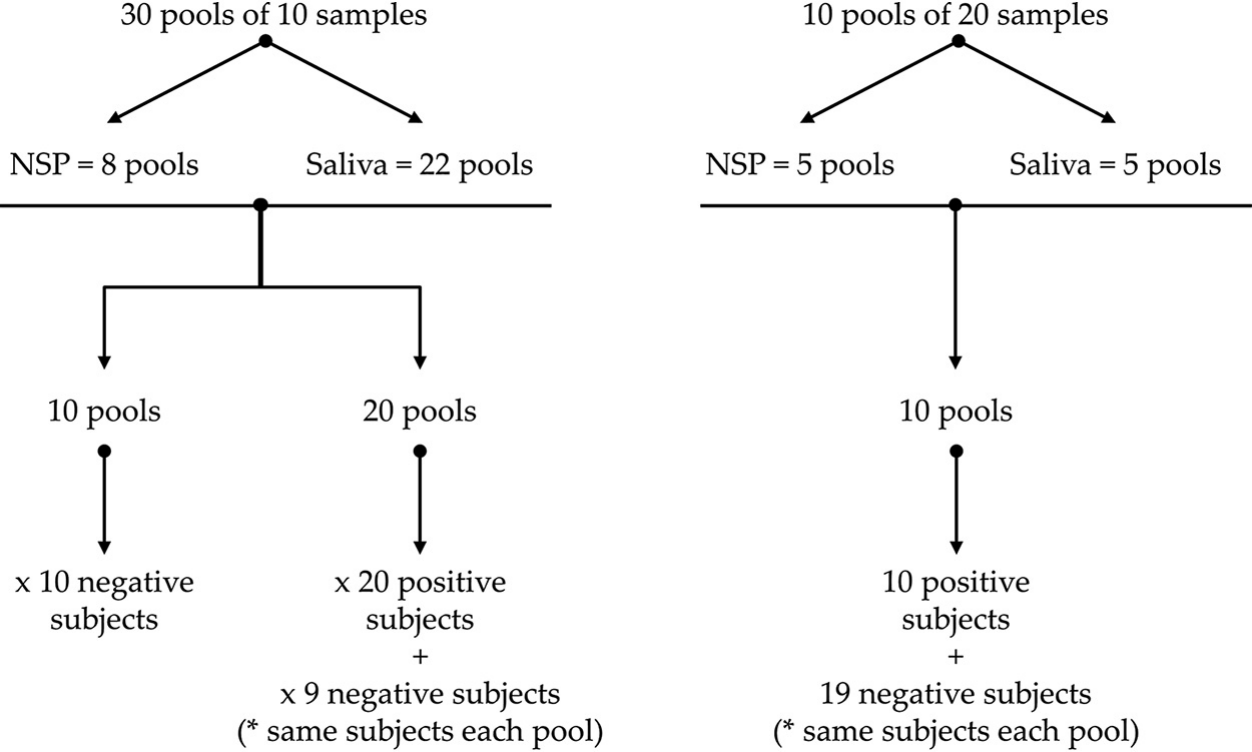
Flowchart for the study.

Samples were combined into pools of 10 samples (*n* = 30 pools) or 20 samples (*n* = 10 pools) (Fig. 3). Before use, samples were eluted with 200 μl phosphate-buffered saline (PBS) (0.9% NaCl; Versylene Fresenius) and stored at −80°C within the Biobank.

Each pool contained equal amounts of one SARS-CoV-2-positive sample (as determined by the Idylla SARS-CoV-2 test; 30 positive samples) and the number of individual SARS-CoV-2-negative samples required to complete the target pool size. RNA extraction from pooled samples and SARS-CoV-2 detection were performed from a total input volume of 200 μl, as recommended by the manufacturer. Thus, 20 μl of each sample was mixed in an Eppendorf tube for a pool of 10 samples, and 10 μl of each sample was mixed for a pool of 20 samples.

### Statistical analysis.

The two-tailed Fisher’s exact test for categorical data was used to analyze associations between variables. The Mann-Whitney nonparametric test was used to analyze the unpaired samples. The kappa agreement test was used for categorical variables. The Pearson product-moment correlation coefficient was used to assess linear dependence between the *C_T_* values. Correlation was judged as follows: very strong, 1 to 0.8; strong, 0.8 to 0.5; fair, 0.5 to 0.2; poor, 0.2 to 0. The normality of data was not tested. The alpha risk was set to 5% (α = 0.05). Statistical analysis was performed with the online software EasyMedStat (version 3.8; EasyMedStat, Neuilly-Sur-Seine; France).
